# Eco-Friendly Jute-Based Hybrid Nonwoven Fabric for Packaging Applications

**DOI:** 10.1021/acsomega.4c07255

**Published:** 2024-10-25

**Authors:** Md. Abdus Shahid, Md. Ahasan Habib, Imam Hossain, Md. Golam Mortuza Limon, Tarikul Islam, Gajanan Bhat

**Affiliations:** † Department of Textile Engineering, Dhaka University of Engineering and Technology, Gazipur 1707, Bangladesh; ‡ Department of Textile Engineering, BGMEA University of Fashion and Technology, Dhaka 1230, Bangladesh; § Department of Textiles, Merchandising and Interiors, University of Georgia, Athens, Georgia 30602, United States; ∥ Department of Textile Engineering, Jashore University of Science and Technology, Jashore 7408, Bangladesh

## Abstract

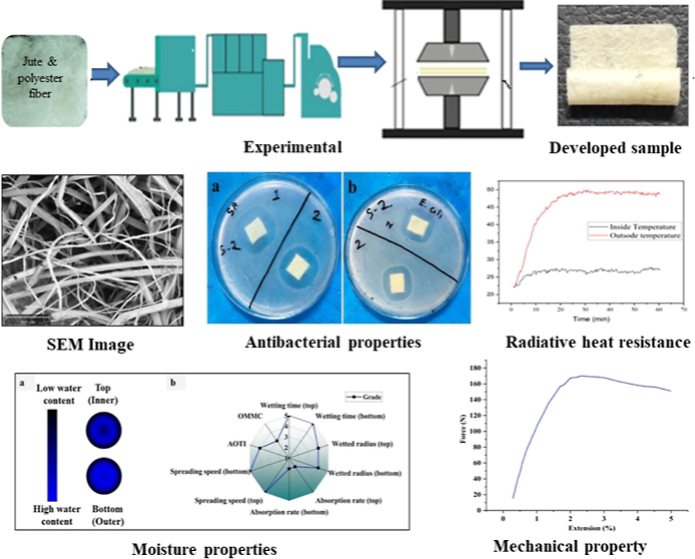

Eco-friendly materials must replace pure synthetic materials to protect the environment and improve human welfare. This study uses a blow room-integrated carding machine to create a filmy web by properly mixing modified jute and polyester fibers. Jute-polyester fiber blended carded webs have been utilized to produce jute-based hybrid nonwoven fabrics (JHNFs), which are subsequently given an antibacterial treatment by spraying a solution of silver nitrate, ethanol, and ammonia. The performance of the developed JHNF is evaluated by its morphological, FTIR, antibacterial, mechanical, and thermal properties. The fiber distribution of jute and polyester fibers is shown in SEM micrographs. The JHNF showed 29 and 27.8 mm ZOI against *Staphylococcus aureus* bacteria and *Escherichia coli*, respectively. The developed sample exhibited a 2.3% extension at the break and 170.8 N of breaking force. The sample confirmed itself as a water-repellent material as water could not penetrate from one surface to another when it was tested by a moisture management tester. The thermal conductivity of the produced sample was measured at 0.0839 W/m K. The lower thermal conductivity signifies a good thermally insulative packaging material. The developed jute polyester nonwoven fabric also demonstrated higher radiative heat resistance, providing a thermal barrier of 22 °C. The porosity, thickness, and density were determined at 49.59%, 0.22 mm, and 0.40 g/cm^3^, respectively. The sample exhibited significant thermal stability, with polymer degradation initiating at temperatures above 280 °C. The developed nonwoven fabric proves its practical utility as a packaging material, offering a viable alternative to pure synthetic packaging materials.

## 1 Introduction

Sustainable packaging describes the development, manufacturing, and application of packaging materials and methods that can lessen their adverse effects on the environment, preserve resources, and promote a circular economy.^1,2^ Across industries, conventional packaging materials such as plastics, metals, glass, and paper-based goods have long served as the foundation for packaging solutions. Traditional packaging materials are widely used. However, they present serious environmental problems, such as pollution, resource depletion, and landfill accumulation. For instance, conventional plastic packaging materials produce a large quantity of garbage; 91% of packaging waste ends up in landfills or the environment. Municipal solid waste primarily consists of plastics, particularly plastics used in packaging. In the category of containers and packaging alone, approximately 14.5 million tons of plastic were produced in 2021. Moving toward more environmentally friendly packaging options that put resource efficiency and environmental responsibility first is necessary to address these issues.^3^

Today, jute is an attractive material for making sustainable packaging materials. Jute is a sustainable raw material for traditional textile products (such as Hessian, carpet backing cloth, sacking, shopping bags, geotextile, and nursery pots), chemical products (such as pulp, microcrystalline cellulose, and carboxymethyl cellulose), and composite products for structural applications (such as beams, plates, and bars). Jute fibers are widely utilized in the textile industry to produce heavy-duty packing materials like gunny sacks and burlap, twine, and rope.^4,5^ Jute fiber is superior to many natural or synthetic fibers in terms of sustainability and mechanical qualities.^6,7^ The greatest effort has been focused on the development of thermosets as structural materials and thermoplastic-based fiber-reinforced composites. By using fewer synthetic polymer ingredients and adding jute fibers to a synthetic polymer matrix could increase its strength.^8^ Many experiments have been conducted on jute bags. Cejudo-Bastante et al. worked to add a naturally occurring antioxidant from the grape pomace extract to jute fibers used in food packaging.^9^ Higazy et al. developed a packaging material incorporating chitosan and jute fabric with antimicrobial properties.^10^ Sengupta and his co-workers worked on nonwoven jute fabric with polyethylene laminated with ethylene vinyl acetate adhesive that can be used for packaging applications.^11^ Shahid et al. investigated how adding microencapsulated phase change material with a hydrophobic binder can improve the thermal and moisture management qualities of jute fabric for packaging applications.^12,13^ Also, kenaf and jute carrier bags have been produced, although they lack antibacterial properties.^14^ Additionally, a flexible nonwoven mat made of poly(vinyl alcohol) and jute has been studied.^6^

Polyester, renowned for being resilient to wrinkling, long-lasting, and low maintenance, rapidly became a mainstay material. Polyester fibers are derived from petroleum-based chemicals by polymerization. The most commonly used monomers are ethylene glycol and terephthalic acid. Because of its strength, resistance to moisture and chemicals, and thermal stability, polyester fiber is used in nontextile industries like packaging, automotive, construction, and geotextiles, in addition to the textile industry. Hamouda and Aly produced a composite material using waste jute fibers and recycled polyester fiber as a reinforcement with a polypropylene matrix for packaging applications.^15^ Habib et al. developed jute-polyethylene nonwoven fabric for packaging applications.^16^

However, environmental concerns have pressured the packaging industry to create sustainable substitutes for conventional materials.^17,18^ There is a need in the market for efficient and environmentally friendly materials. Yet, current solutions frequently fall short of balancing sustainability and functional performance. This study fills this vacuum by investigating the possibilities of hybrid nonwoven textiles based on jute as a workable option for packaging applications. This study offers a novel material that satisfies the functional requirements of packaging and promotes environmental sustainability by blending jute with other fibers. By proving that utilizing natural fibers in hybrid designs is feasible, this work advances knowledge in industry and opens the door for future advancements in environmentally friendly packaging materials. The results could have an impact on industrial processes as well as scholarly studies, promoting the wider use of environmentally friendly products. Therefore, the primary objective of this study is to fabricate a jute-based hybrid nonwoven fabric (JHNF) and comprehensively assess its morphological, mechanical, and thermal attributes to ascertain its suitability for packaging applications.

## 2 Materials and Methods

### 2.1 Materials

Jute (Tossa) fiber and polyester fiber were sourced from a local market in Dhaka, Bangladesh. Lab-grade NaOH and H_2_O_2_ were gathered from Merck, Germany. Ethanol, ammonia, and silver nitrate were sourced from the Jonaki Scientific Store on Hatkhola Road, Dhaka. Since all of the sourcing chemicals were of analytical reagent grade, they could all be used without additional purification.

### 2.2 Methods

#### 2.2.1 Preparation of Jute-Polyester Carded Web

The collected jute fiber was scoured with 5% wt. NaOH. After that, scoured jute was bleached for 45 min at 80 °C with 3% wt. H_2_O_2_. Jute and polyester fibers were properly mixed in a ratio of 70:30 and fed into the blow room-integrated carding equipment. Finally, the carded web—a mixture of polyester and jute fibers—was collected from the carding machine.

#### 2.2.2 Preparation of Jute-Based Hybrid Nonwoven Fabric

**Figure 1:**
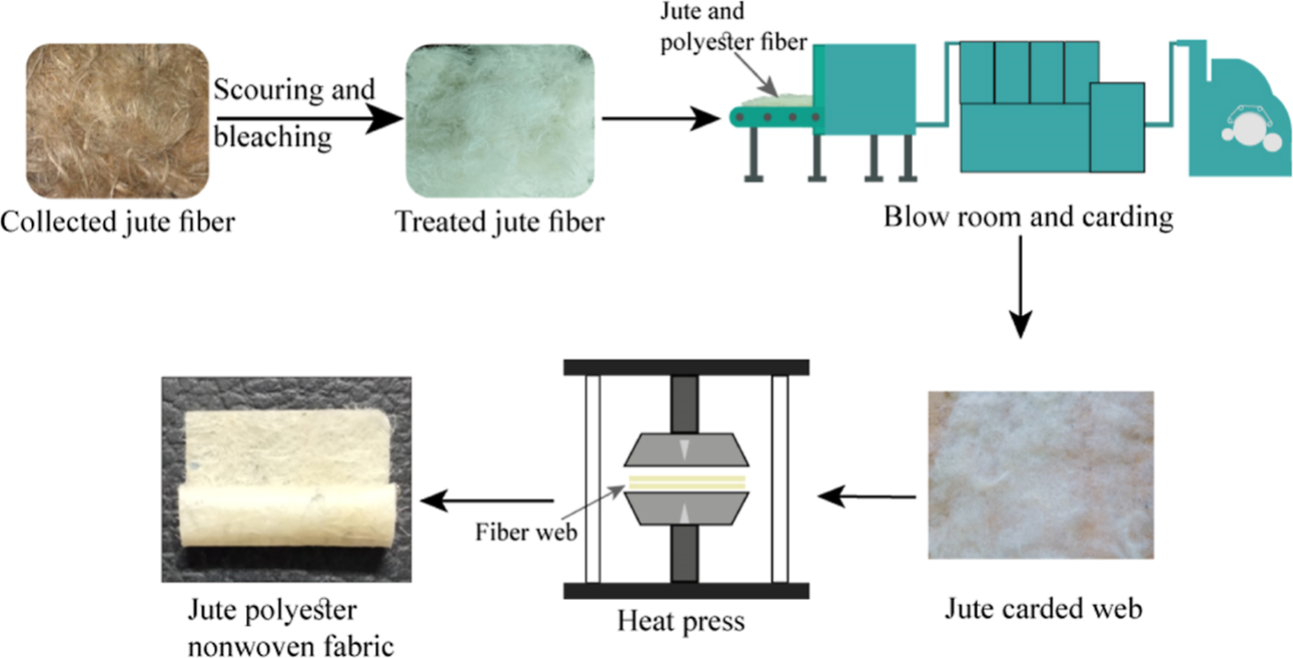
Schematic diagram to fabricate the JHNF.

The carded jute-polyester web was securely placed on the heat-pressing machine’s lower plate. As a result, the nonwoven jute web was covered with a polyester film. Subsequently, the fluffy jute web and the polyester film were pressed for 7 min at 150 °C with a five-ton load using a heat press machine. By applying pressure, the polyester fiber melts and forms bond points with the jute fiber, providing structural integrity (see Figure 1). The generated JHNF was sprayed with a solution of ethanol, ammonia, and silver nitrate to impart antibacterial properties. It was then allowed to dry at room temperature for 4 h.

#### 2.2.3 Characterizations

The morphological orientation of the nonwoven fabric was observed using a scanning electron microscope (SU-1510, Hitachi, Japan) at magnifications of 3000 and 5000. The voltage used was 5 kV. In addition to calculating the porosity of the sample, ImageJ software was used.

The chemical structures of the jute fibers, polyester fiber, chemicals, and jute nonwoven with chemical samples were characterized by Fourier transform infrared (FTIR) spectroscopy (IRPrestige21, Shimadzu Corporation, Japan). The sample spectra were obtained in the 500–4000 cm^–1^ region at a resolution of 4 cm^–1^.

The disc diffusion method against *Staphylococcus aureus* and *Escherichia coli* bacteria confirmed the antibacterial ability of samples by using the SILVADUR 930 antibacterial agent. The zone of inhibition was determined. The bacteria were present on 6 mm diameter disks at a concentration of 1.5 × 10^5^ CFU/mL.

ISO 9073-2:1995 is used to measure the thickness of the nonwoven fabrics. A thickness gauge with a flat circular presser foot is used to apply a predetermined pressure of 2 kPa on the fabric. The thickness is measured many times throughout the fabric to get an average result.

The mass per unit area is calculated by cutting and precisely weighing a fabric sample (usually 100 cm^2^) using the standard procedure ISO 9073-1:1989. To get the mass per unit area, we divided this weight by the area (see eq 1). The mass per unit area of nonwoven fabrics is divided by the fabric’s thickness to determine their density.^19^ Ultimately, the density is computed as follows
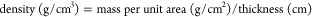
1

Using a universal strength tester (Testometric M250-3CT), the breaking force and extension percentage at the break of the JHNF were assessed following ASTM D5034. An extension rate of 300 mm/min was used. It was measured by 15 × 10 cm.

The moisture management tester (MMT) (M290, SDL Atlas, UK) was used to investigate the behavior of the developed nonwoven fabric by AATCC 195-2009. This determines the moisture (liquid) in terms of absorption rate, wetting time, spreading speed of the outer and inner surfaces, maximum wetted radius, accumulative one-way transport capacity (R), and overall moisture management capacity.^12^

The produced thermal conductivity of the fabric was assessed using a two-plate method in a steady-state setting, in accordance with standard BS 4745:2005. JHNF’s radiative heat resistance was evaluated by placing a sample 20 cm from a 25 W incandescent bulb. The experiment used a masking tape to shield the temperature sensors appropriately. Heat sensors were utilized for 60 min to track the temperature increase of the surface exposed to radiative heat. The opposite surface was exposed to the surrounding air at 30 s intervals. The ELTRA Thermostep 12ML (88100-3010) thermogravimetric analyzer was used to assess the thermal degradation under ASTM D7348-08e1 with a heating rate of 20 °C/min.

## 3 Results and Discussion

### 3.1 Morphological Analysis

**Figure 2:**
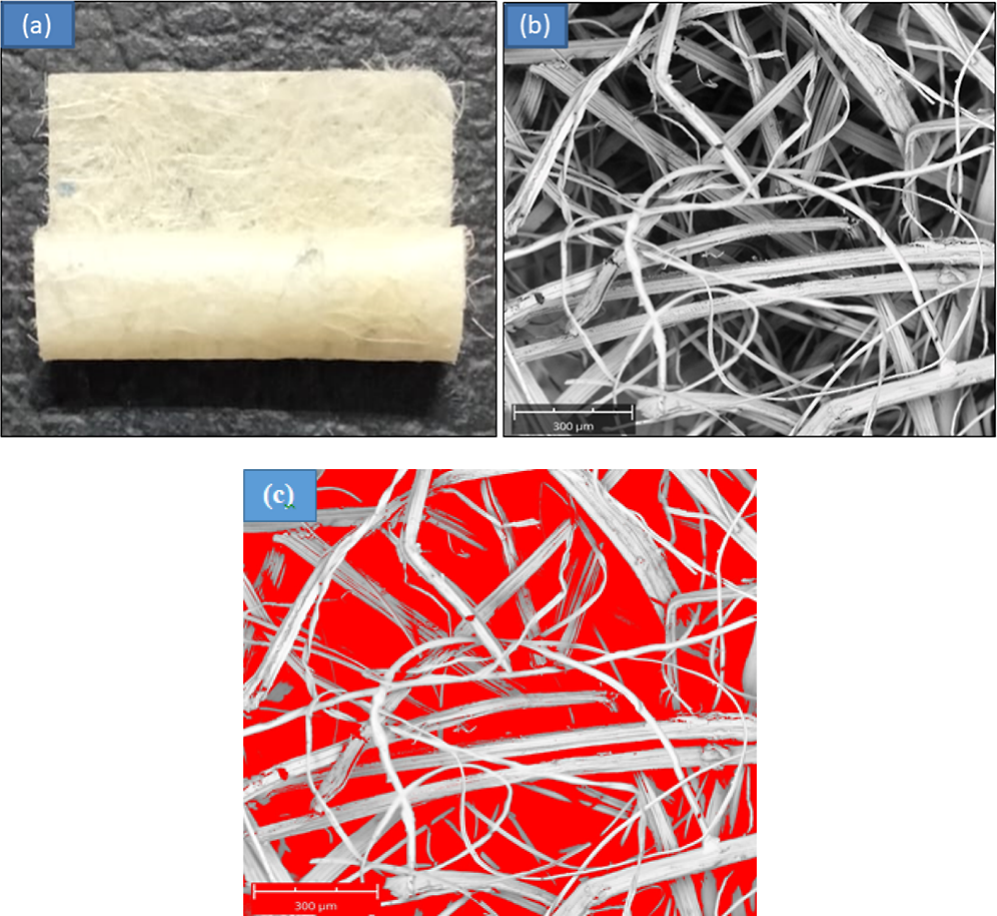
(a) Actual image, (b) SEM image, and (c) porosity of developed JHNF.

SEM analysis enables the examination of fiber organization, surface morphology, and homogeneous interfacial adhesion between jute and polyester fibers in the developed sample. This performs comprehensive visual and structural analysis of the sample. All fibers are attached in a random manner. Understanding the material’s microstructure is made more accessible by this analysis, which also affects the mechanical qualities, performance traits, and applicability of the material for different applications. SEM and actual images of the sample are displayed in Figure 2. The calculated porosity was 49.6%, which is suitable for packaging material.

### 3.2 FTIR Analysis

**Figure 3:**
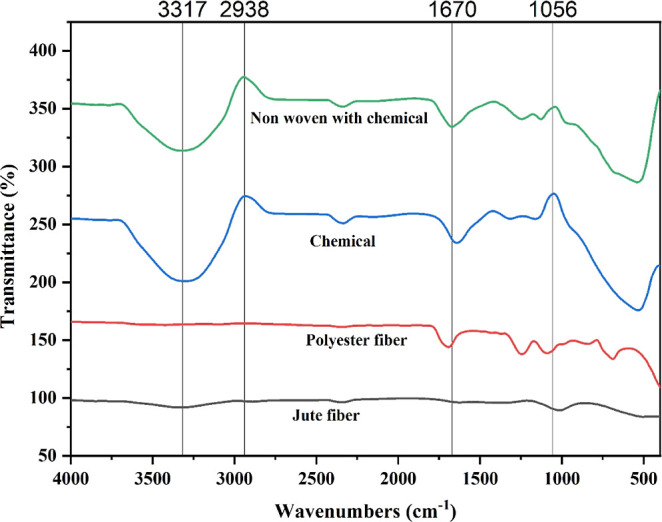
FTIR spectra of jute, polyester, antimicrobial chemical, and developed samples.

FTIR was used to determine the chemical bonds and functional groups that confirm the presence of a material in a sample. The FTIR spectra (Figure 3) showed peaks at 3317 and 1056 cm^–1^ wavenumbers. Jute showed an extensive peak around 3600–3200 cm^–1^ and peaks around 1600–1300, 1200–1000, and 800–600 cm^–1^. These peaks indicated the presence of hydrate and hydroxyl (OH−) in the cellulose and hemicellulose of the jute fiber.^20^ Polyester fiber showed peaks at 1670 and 1056 cm^–1^, the bond vibrations at 1700 cm^–1^ –C=O, the aromatic ring at 1200 cm^–1^, and the O=C–O–C at 1100 cm^–1^.^21^ The chemical showed peaks at 2938 and 1056 cm^–1^ due to the presence of AgNO_3_^22^ and showed peaks at 3317 and 1700 cm^–1^ due to the presence of ethanol^23^ in this antibacterial agent. Similar peaks in nonwoven samples confirmed the presence of jute fiber, polyester fiber, and chemicals, confirming the presence of all the materials as expected.

### 3.3 Antibacterial Properties

**Figure 4:**
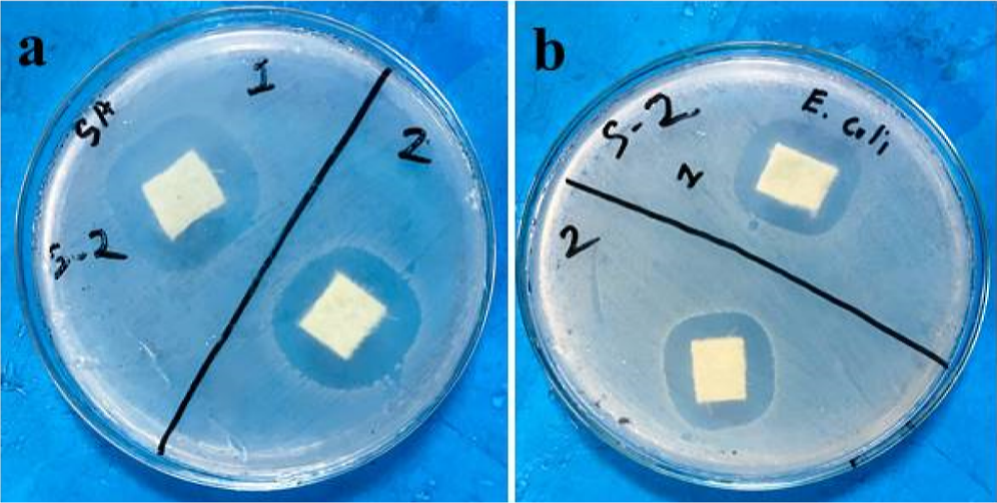
Antibacterial performance of JHNF against (a) *S. aureus* and (b) *E. coli*.

The antibacterial properties of nonwoven jute packaging materials are necessary for packaging applications. That is why the antibacterial properties of JHNF samples were evaluated against *S. aureus* and *E. coli*, and the results are shown in Figure 4. The samples incorporated with antibacterial chemicals showed 29 and 27.8 mm ZOI against *S. aureus* bacteria and *E. coli*, respectively. The presence of silver nitrate, which successfully reduced bacterial growth, is responsible for the antibacterial efficacy of these samples.^24^ This sample is ideally suited for use in food packaging materials due to its demonstrated antibacterial performance.

### 3.4 Thickness

For packing applications, nonwoven material thickness is important since it affects the overall performance, mechanical strength, durability, and barrier qualities of the material. The thickness of the JHNF sample was 0.22 mm and showed good uniformity. Consistent thickness helps satisfy industrial standards and legal requirements while preserving quality control, maximizing resource utilization, and achieving cost-effectiveness. A well-balanced thickness guarantees that the material offers sufficient protection and pliability, improving the packaging’s usability and visual attractiveness.

### 3.5 Density

Bulk density is crucial for nonwoven materials used in packing because it provides insight into the material’s structural characteristics, including porosity, compressibility, and weight distribution. A denser, more durable material with improved load-bearing capacity and protective properties is generally indicated by a larger bulk density, which makes it appropriate for packing bulkier or more delicate goods. By measurement of the mass and volume of the JHNF sample, the density was determined to be 0.40 g/cm^3^.

### 3.6 Mechanical Behavior

**Figure 5:**
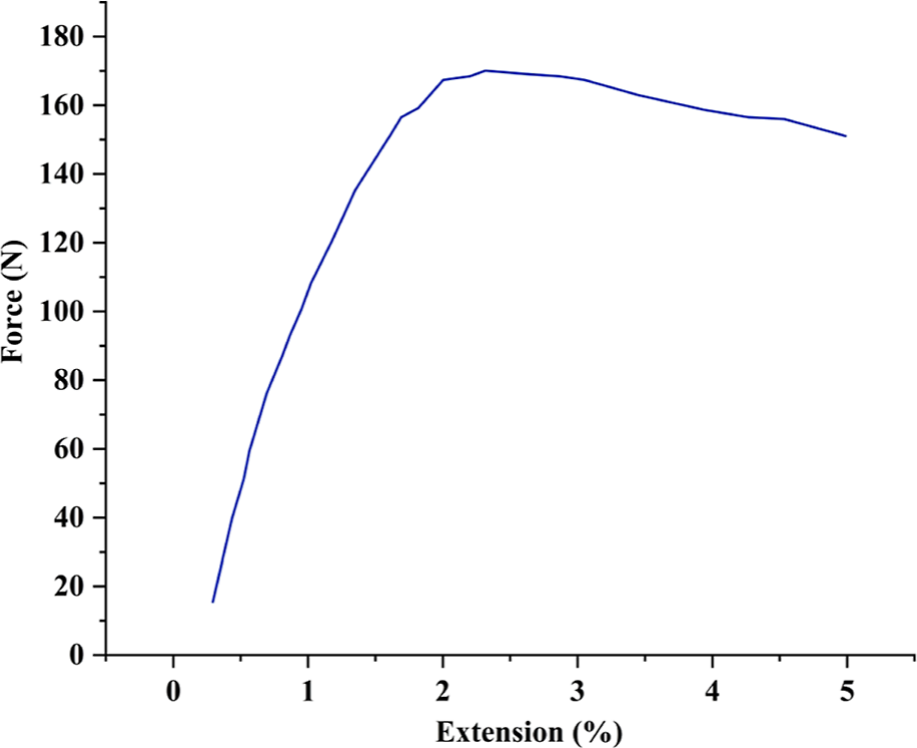
Force–extension curve of JHNF.

The maximum load a nonwoven fabric can bear before breaking is determined by tensile testing. When evaluating a material’s ability to withstand stresses and forces from its surroundings, tensile strength is an essential parameter. For packaging materials, it is essential that the fabric can endure a load without tearing or breaking. Figure 5 shows the JHNF tensile properties. The breaking force and elongation (%) were evaluated. The JHNF showed 2.3% extension at break and a higher breaking force of 170.8 N. The maximum stress a material can sustain before deforming when stretched or tugged is indicated by its tensile strength, which is an essential characteristic. Tensile strength, calculated with thickness and width considered, results in a torque of 26.89 MPa. The tensile strength of a bundle of jute fibers is around 71 MPa,^25^ and, in the case of polyester, it is around 35 MPa.^26^ Thus, the prepared JHNF sample demonstrated a suitable tensile strength, making it an effective option for packing material for handling or carrying. A uniform nonwoven web guarantees even dispersion to withstand the force of jute fibers. The contribution of fiber properties and the structure of the fabric affects the tensile properties. The combination of good strength and elongation indicates a well-bonded nonwoven structure that shows good realization of fiber properties.

### 3.7 Moisture Management Properties

**Figure 6:**
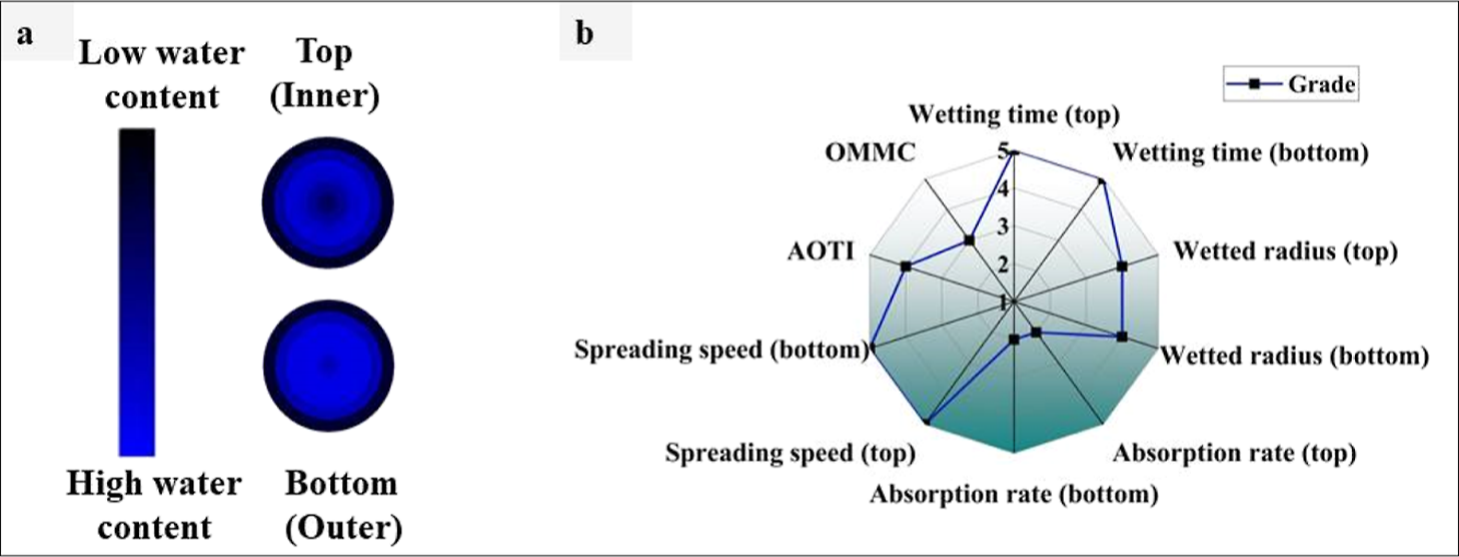
Moisture management (a) profiles and (b) grading of the JHNF sample.

The nonwoven packing capacity of material to effectively transport moisture away from the surface, a critical function for preserving product integrity and averting moisture-related damage, is assessed by the MMT. Through the quantification of moisture transport properties of a material in controlled settings, the MMT offers significant insights for improving the moisture management performance through packaging design and material selection optimization. The results of JHNF’s moisture management profile are shown in the radar chart (see Figure 6b). The JHNF took 5.8 s to wet the inner side and 13.4 s to soak the outside side. The wetted radius, spreading speed, and absorption rate were 5 mm, 0.82% per second, and 6.04 mm/s, respectively, for the top surface. The one-way transport capability of the sample was 0.46. This test considered the fabric to be a moisture management fabric. The water location graphic illustrates how much moisture moves through the inner surface of the material based on these measurements (see Figure 6a). Jute is very moisture absorbing and hydrophilic in nature,^28^ and polyester is hydrophobic in nature;^27^ therefore, the developed sample exhibited moisture management fabric.

### 3.8 Thermal Stability

**Figure 7:**
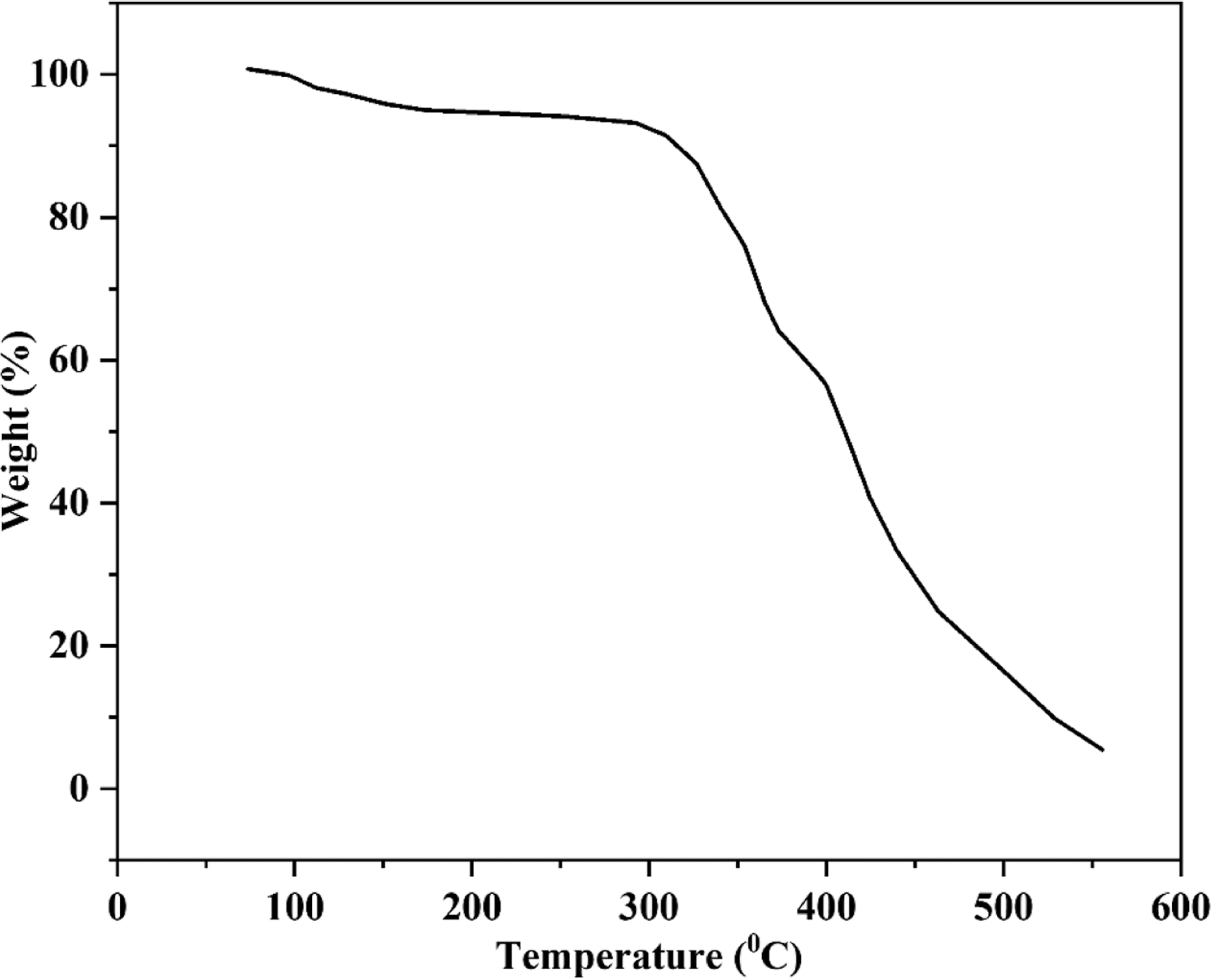
TGA curve of JHNF.

By monitoring weight loss as a function of temperature, the TGA test for nonwoven fabric assesses its thermal stability and decomposition behavior. When determining whether a material is appropriate for uses that involve exposure to high temperatures, like packaging, the main component that initiated the weight loss was water at a temperature range of 100–280 °C. Polyester has a melting point between 115 and 145 °C, but jute fiber degrades thermally at temperatures above 200 °C. Polymer degradation occurred in the temperature range of 280–420 °C,^28,29^ as presented in Figure 7. The main weight loss occurred in this range. The interaction between jute and polyester, which restricted segmental motion and hindered the movement of polymer chains, was responsible for the increased stability. The highest temperature at which new structures formed due to polymer degradation was recorded at 280 °C. As a result of these polymeric components’ carbonization process, weight loss followed. The above result confirmed the usability of the sample in terms of the thermal stability performance.

### 3.9 Thermal Conductivity

The thermal barrier qualities of the jute fiber are remarkable. Jute fiber has a thermal conductivity of 0.038–0.040 W/m K.^16^ Its peculiar cellular structure and low density are among the unique natural properties linked to this.^30^ Conversely, polyester has a thermal conductivity of 0.157 W/m K.^31^ The thermal conductivity of JHNF was measured at 0.084 W/m K. Jute fibers have low density and cellular structure, resulting in low heat conductivity, which enhances their ability to act as a thermal barrier. However, JHNF has a slightly higher thermal conductivity due to the increased thermal conductivity of polyester. The porosity of the fabric also contributes to a reduction in the thermal conductivity of the fabric compared to that of the neat fiber. Reduced thermal conductivity is advantageous for maintaining stable temperatures for products sensitive to heat or cold. During their storage and transport, materials can be unaffected by hot and cold environmental conditions.

### 3.10 Radiative Heat Resistance

**Figure 8:**
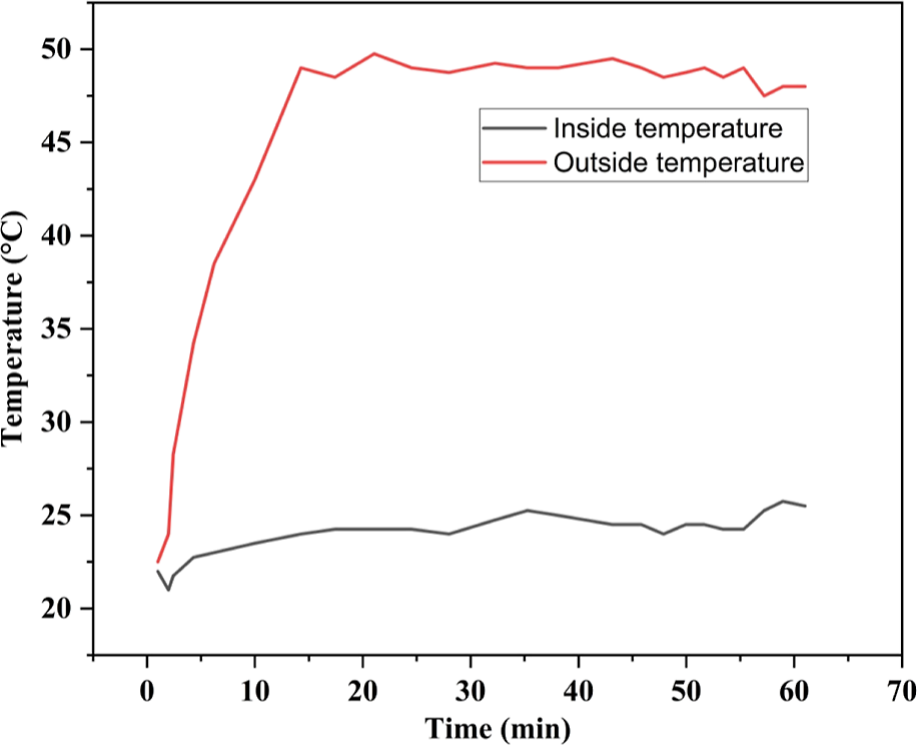
Radiative heat resistance curve of JHNF.

The radiative heat resistance performance was assessed by exposing the samples to a radiative heat source and measuring the outer surface temperature to gauge the heat transfer through the sample. As depicted in Figure 8, the time–temperature curve shows a gradual increase in the inner and outer surface temperatures of the JHNF sample over time. The sample demonstrated strong resistance to radiative heat due to the low thermal conductivity of the jute fiber and polyester. At the end of the experiment, JHNF exhibited a surface temperature of 48.50 °C when exposed to light, while the opposite surface temperature was 26.50 °C. The experiment revealed a significant contrast in the time–temperature curves of the opposite surface, with a maximum difference of 22 °C for JHNF at the conclusion of the test. Thus, the JHNF sample is demonstrated to be suitable for use as a radiative heat-resistant material in packaging applications.

## 4 Conclusions

In this work, JHNF development and its application as a packaging material are envisaged. This study aimed to produce the nonwoven fabric by carding and employing heat pressing. The developed nonwoven fabric was characterized by its morphological structure, FTIR, antibacterial, mechanical characteristics, moisture management properties, thermal characteristics, and physical attributes (density, thickness, and porosity). The developed samples showed a sufficient antibacterial performance. The developed nonwoven fabric is flexible enough to be utilized as a packaging material. It demonstrated high breaking force and extension at the break, which are essential for packaging material. The developed JHNF also possessed water-repellent characteristics, which are crucial for the packaging material. It exhibited lower thermal conductivity, radiative resistance, and higher thermal stability. Materials in a warm state are often easier to transport without incurring damage. This material can be a good choice for packing temperature-sensitive items because of its low thermal conductivity and high thermal stability. The developed material is more useful in conditions where moisture resistance is essential, such as food packaging, because of its water-repellent qualities. Packaging materials in industries where hygiene is crucial can greatly benefit from the additional layer of protection provided by the proven antibacterial qualities. However, investigating other natural fibers or biodegradable polymers with jute could yield an even more adaptable and sustainable material. Moreover, adding other antimicrobial agents to the antibacterial treatment or putting them straight into the fiber matrix may improve the barrier abilities of fabric and make it applicable for a wider range of packaging uses, especially in the food and medical sectors. The developed fabric contains a small amount of polyester, thereby limiting the use of thermoplastic materials in daily applications.
